# Supplementary material for: In vivo nuclear capture and molecular profiling identifies Gmeb1 as a transcriptional regulator essential for dopamine neuron function
Source: Nat Commun. 2019 Jun 7;10:2508. doi: 10.1038/s41467-019-10267-0 (PMC6555850; doi:10.1038/s41467-019-10267-0)
Supplement: Supplementary file 1 — Supplementary Information [file 41467_2019_10267_MOESM1_ESM.pdf]

In vivo nuclear capture and molecular profiling identifies *Gmeb1* as a transcriptional regulator essential for dopamine neuron function

Corresponding author: Yi Zhang

Supplementary Figures

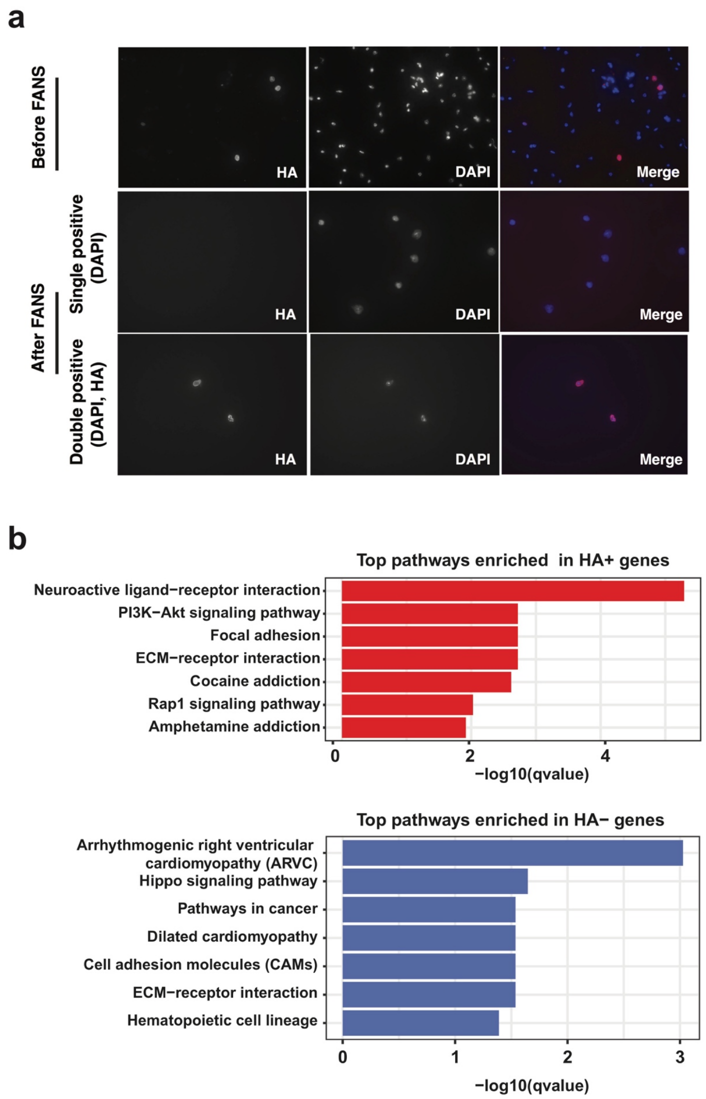

Supplementary Figure 1 | (Related to Figure 1) FANS sorting of HA-tagged mDA nuclei and pathway enrichment analysis

- **(a)** FANS sorting results: Upper panel, midbrain nuclear mixture before FANS sorting – 20x micrographs showing HA^+ nuclei (fluorescence immnunostained, left), DAPI+ nuclei (middle), and merged view (right). Middle panel, single-positive ($HA^-/DAPI^+$) nuclei following FANS – 40x micrographs show negative signal for HA (left), positive signal for nuclei (middle), and merged view (right). Bottom panel, double-positive ($HA^+/DAPI^+$) nuclei following FANS – 40x micrographs show positive signal for HA (left), positive signal for nuclei (middle), and merged view showing colocalization of signal (right).
- **(b)** KEGG pathway enrichment of HA^+ and HA^- cells (*q-value*<0.05).

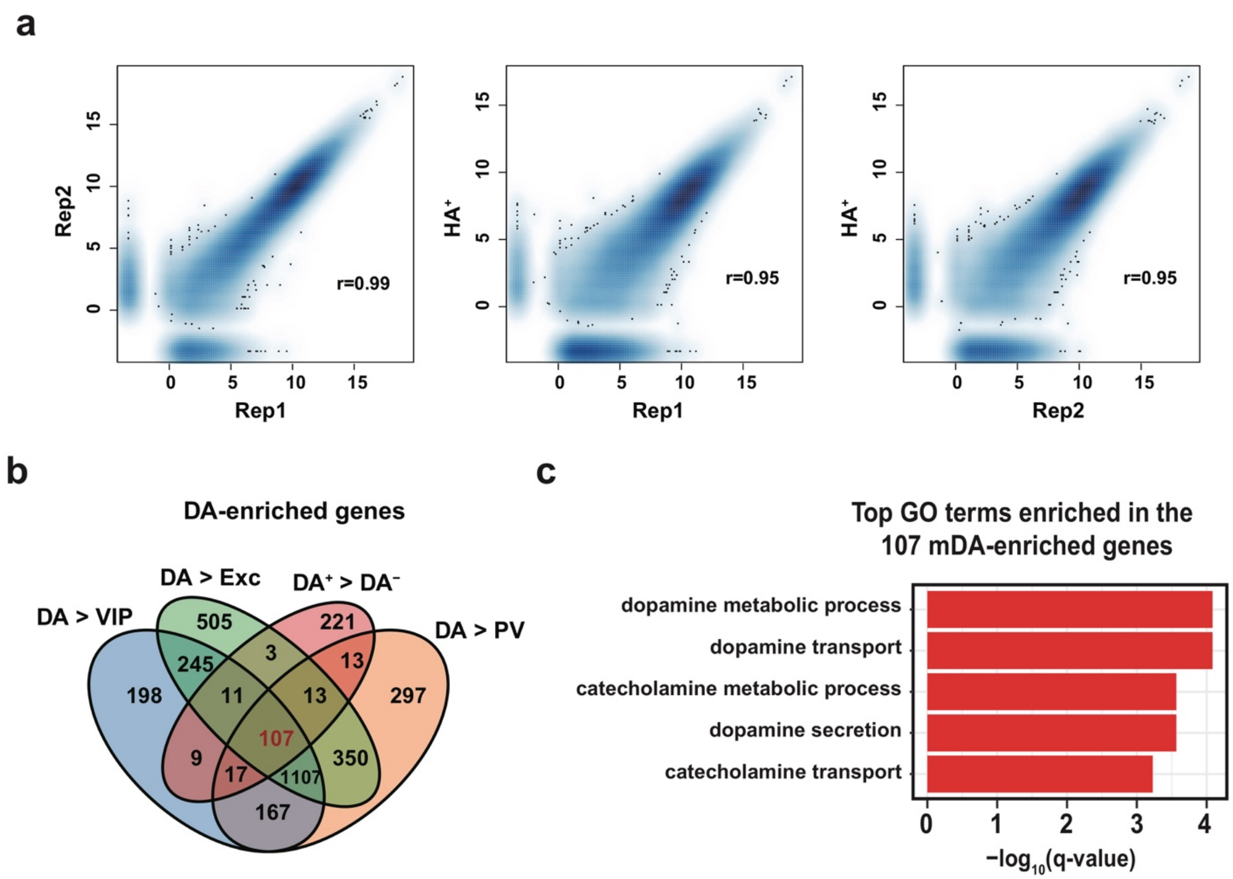

Supplementary Figure 2 | (Related to Figure 2) mDA neuron transcriptome analysis

- **(a)** Scatter plot showing the R-squared correlation between the two mDA neuron samples and the HA+ RNA-Seq data
- **(b)** Venn diagram showing the number of genes enriched in mDA neurons compared to those in cortical neuron subtypes (DESeq2, *q-value* < 0.001 and FC >4). 107 genes were highly expressed in mDA neurons compared to PV-, VIP-expressing and excitatory cortical neurons.
- **(c)** KEGG pathway enrichment of the 107 mDA neuron-enriched genes (*q-value* < 0.05).

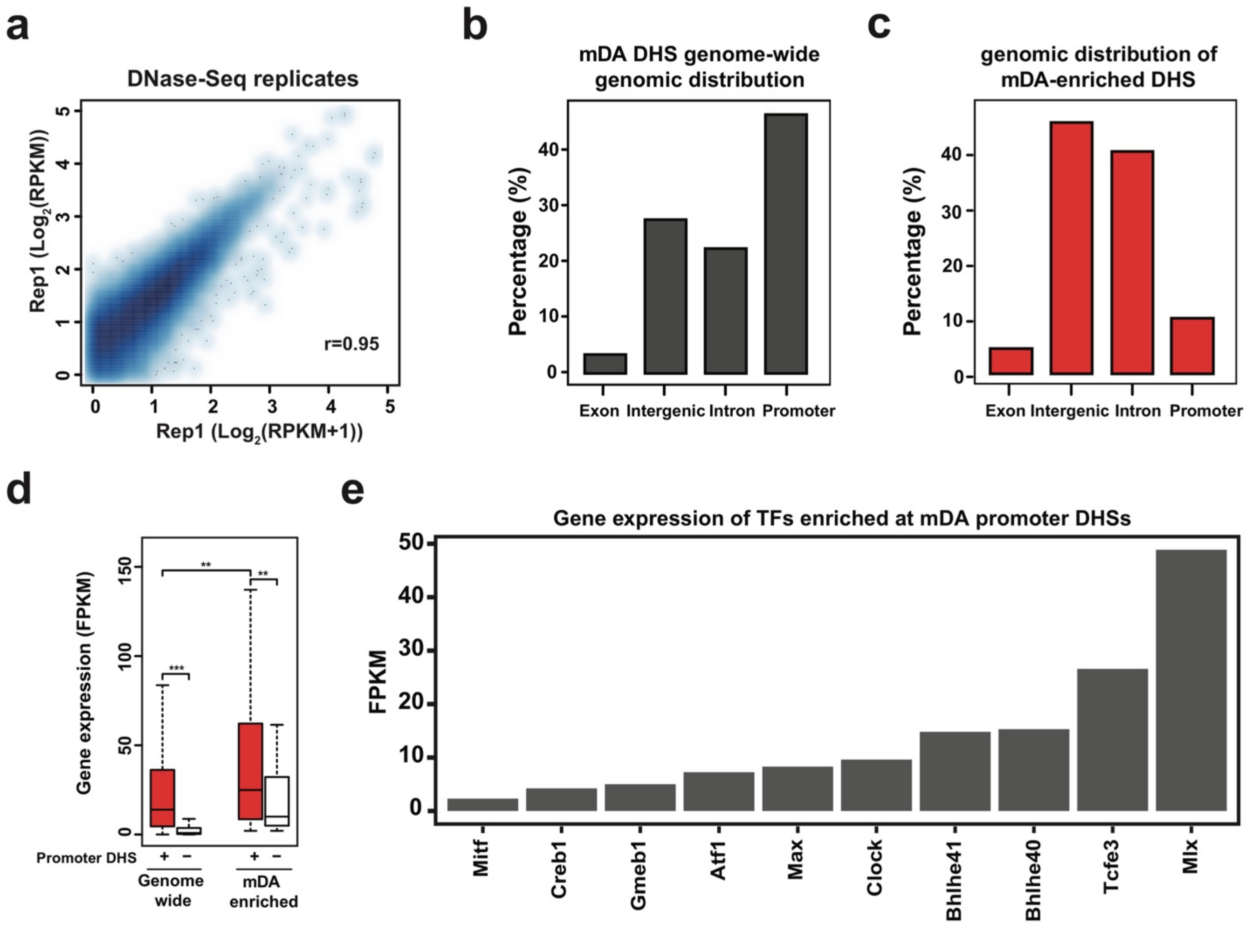

Supplementary Figure 3 | (Related to Figure 2) mDA neuron promoter DHS analysis

- **(a)** Scatter plot showing the correlation between DHS peaks $\log_2$(RPKM+1) of the two mDA liDNase-Seq replicates (Pearson Coefficient: 0.95).
- **(b)** Bar graph showing distribution of the 28,084 mDA DHSs across genomic features.
- **(c)** Bar graph showing distribution of the 2,374 mDA-enriched DHS across genomic features.
- **(d)** Boxplot showing genes with a promoter DHS are expressed at a higher level (FPKM) compared to those without promoter DHS. This is true at the genome-wide scale or for the 107 mDA-enriched genes (****p-value*= 2.2e-16 and ***p-value*= 0.005397, respectively. Mann-Whitney-Wilcoxon Test).
- **(e)** Expression level (FPKM) of the 11 TFs with binding motifs at the promoter DHS of mDA-enriched genes.

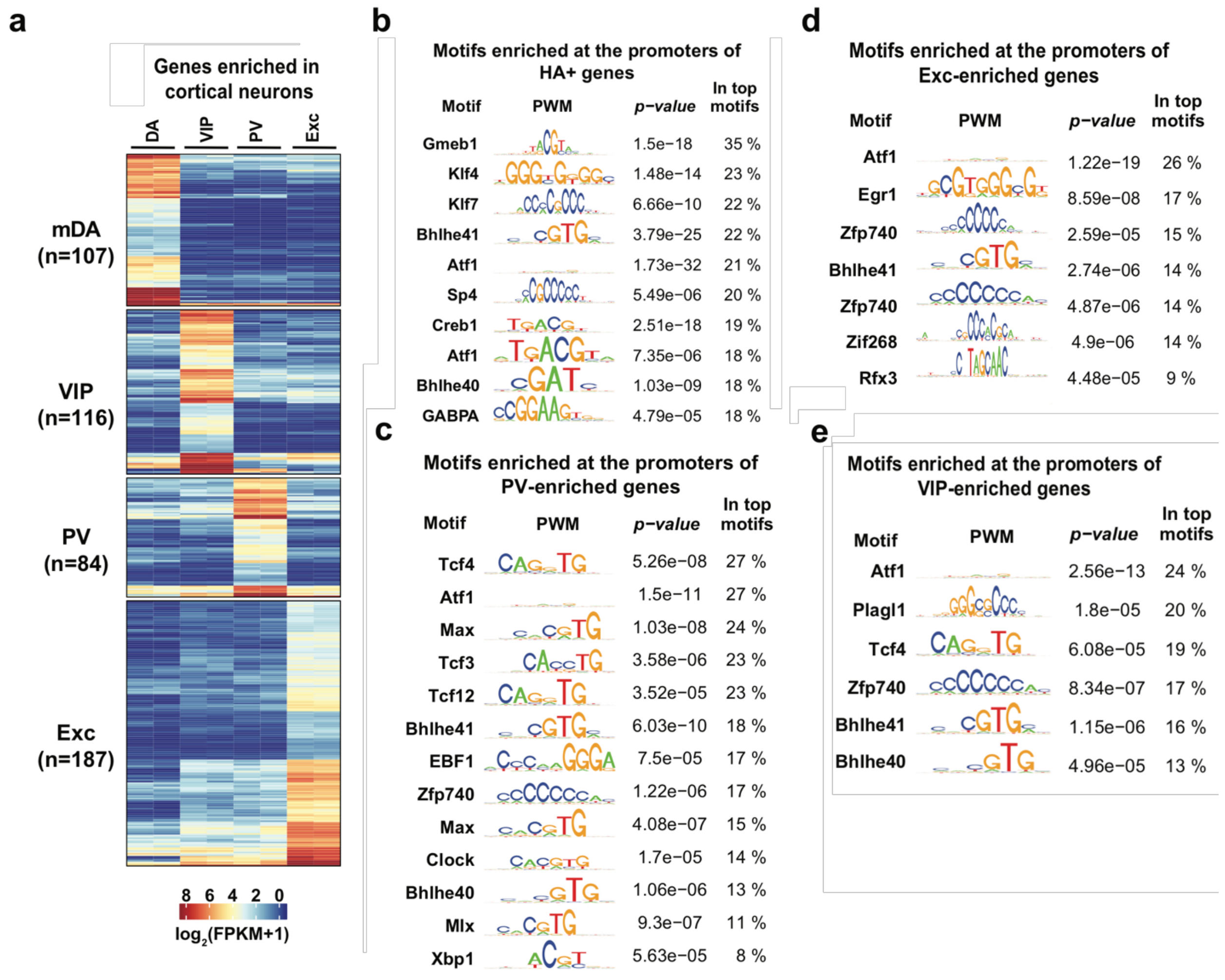

Supplementary Figure 4 | (Related to Figure 2) Motif analysis of cortical neuron-enriched genes

- **(a)** Heatmap showing the log2 expression of genes enriched between mDA and the three types of cortical neurons VIP/PV/Exc (*q-value* < 0.001, FC > 4 and FPKM >1 in the main cell type).
- **(b)** List of motifs identified at the promoters of the 394 HA+ enriched genes (*p-value* < 0.0001, FPKM>1).
- **(c)** List of motifs identified at the ATAC-Seq promoter peaks of PV-enriched genes (*p-value* < 0.0001, FPKM>1).
- **(d)** List of motifs identified at the ATAC-Seq promoter peaks of Exc-enriched genes (*p-value* < 0.0001, FPKM>1).
- **(e)** List of motifs identified at the ATAC-Seq promoter peaks of VIP-enriched genes (*p-value* < 0.0001, FPKM>1).

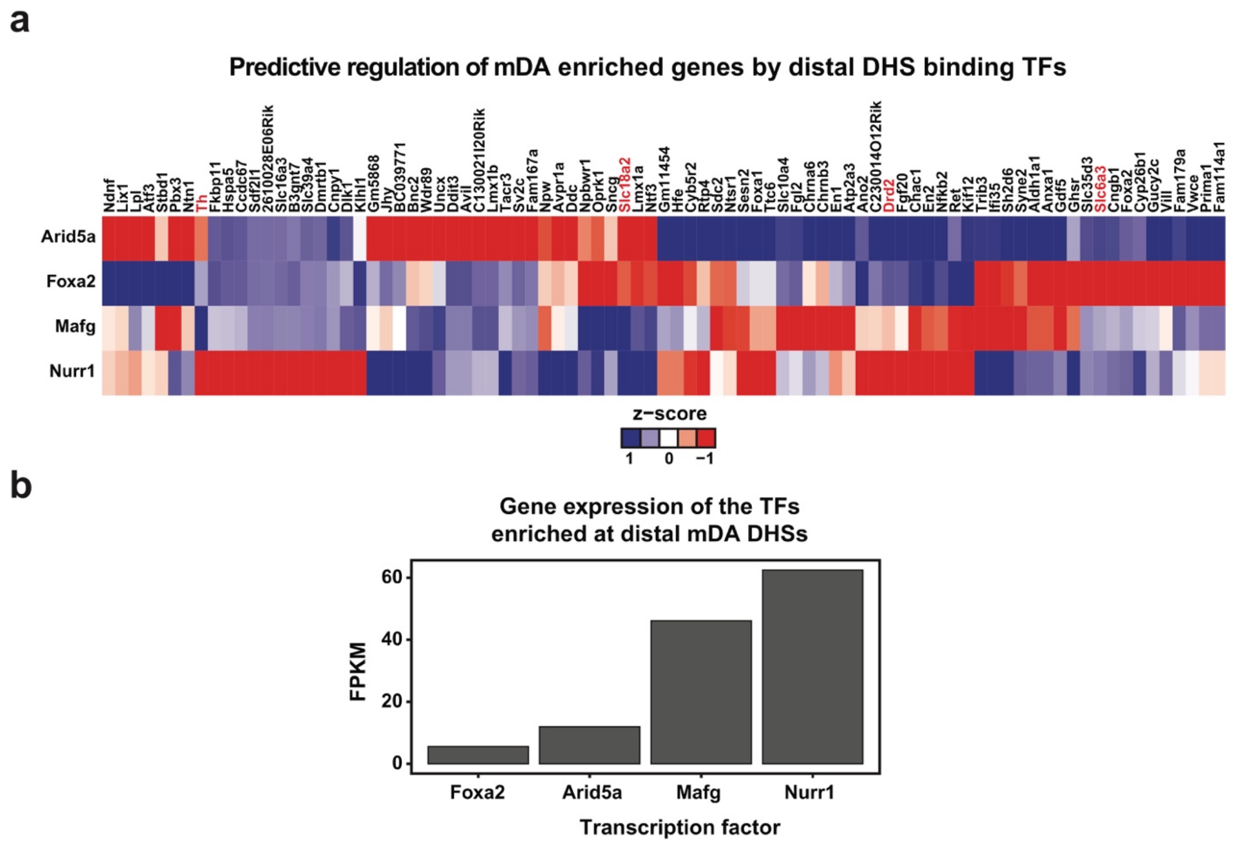

Supplementary Figure 5 | (Related to Figure 3) mDA neurons distal regulator analysis

- **(a)** Heatmap showing the normalized regulatory scores of the identified distal regulators (showed in columns) to the 85 mDA-enriched genes with an mDA-enriched distal DHS.
- **(b)** Bar plot showing the gene expression of the identified distal regulators in mDA neurons.

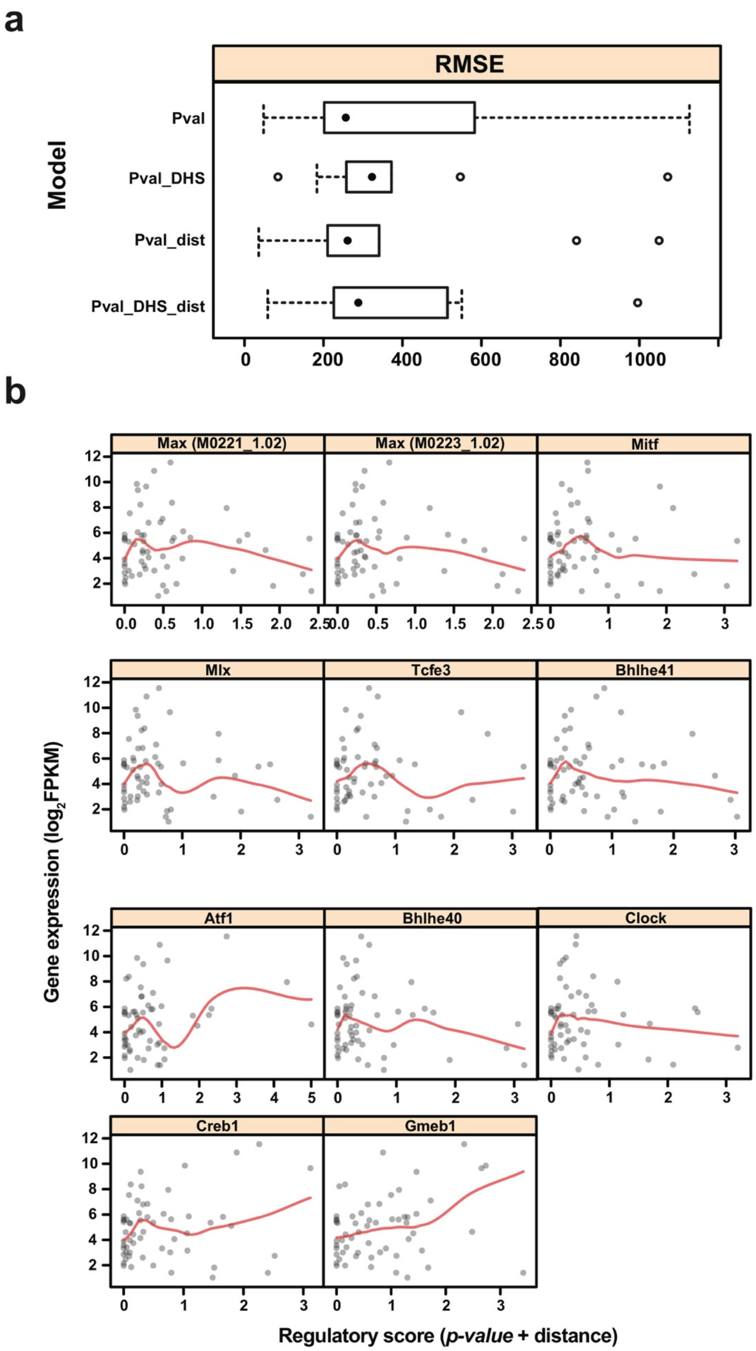

Supplementary Figure 6 | (Related to Figure 3) TF-gene pair predictive model

- **(a)** Box-plots showing the distribution of the root mean square errors (RMSE) of the different TF regulatory score models in predicting the gene expression of the 59 mDA-enriched genes with a promoter DHS. We noticed that while motif binding *p-value* alone did not correlate well with gene expression, combining the motif binding *p-value* and the distance to TSS yielded a better prediction.
- **(b)** Scatterplots showing the correlation of the "*p-value* + distance to TSS" regulatory score for each of the 11 TFs identified at the promoter DHS of mDA-enriched genes with the expression of the 59 mDA-enriched genes with a promoter DHS.

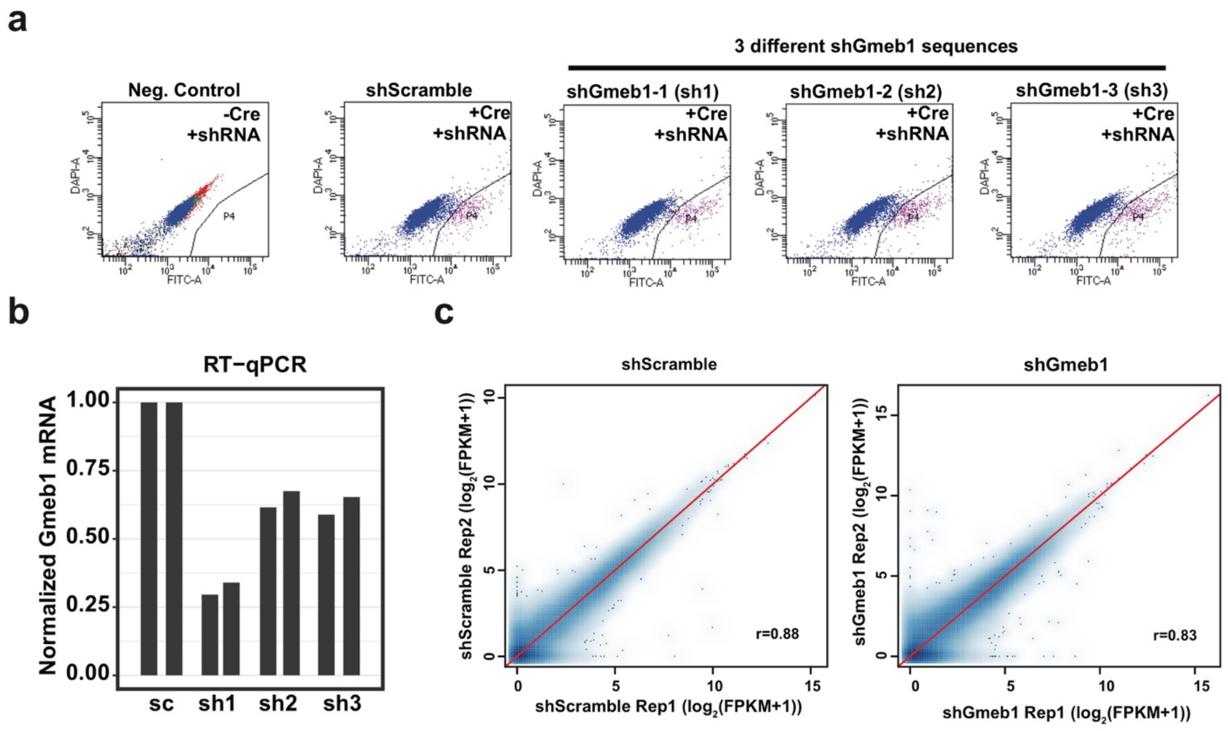

Supplementary Figure 7 | (Related to Figure 3) shRNA testing and RNA-Seq data reproducibility

- **(a)** FACS scatterplot of N2A cells transfected with Cre-recombinase (Cre) and DIO-KASH-GFP-U6-shRNA (shRNA). Negative control (left) showing lack of GFP-containing cells (P4 section of plot), while groups transfected with Cre-recombinase produced GFP-containing cells, suggesting that expression of KASH-GFP is Cre-inducible. N2A cells expressing GFP were FACS sorted for RT-qPCR.
- **(b)** RT-qPCR results showing that shGmeb1 sequence 1 (sh1) reduces *Gmeb1* mRNA in N2A cells to about 30% that of the shScrmable (Sc) control. Two biological replicates were analyzed for each condition.
- **(c)** Scatter plot showing the correlation of the RNA-Seq results between the two biological replicates of shScramble (Pearson coefficient: 0.88) and shGmeb1 (Pearson coefficient: 0.83).

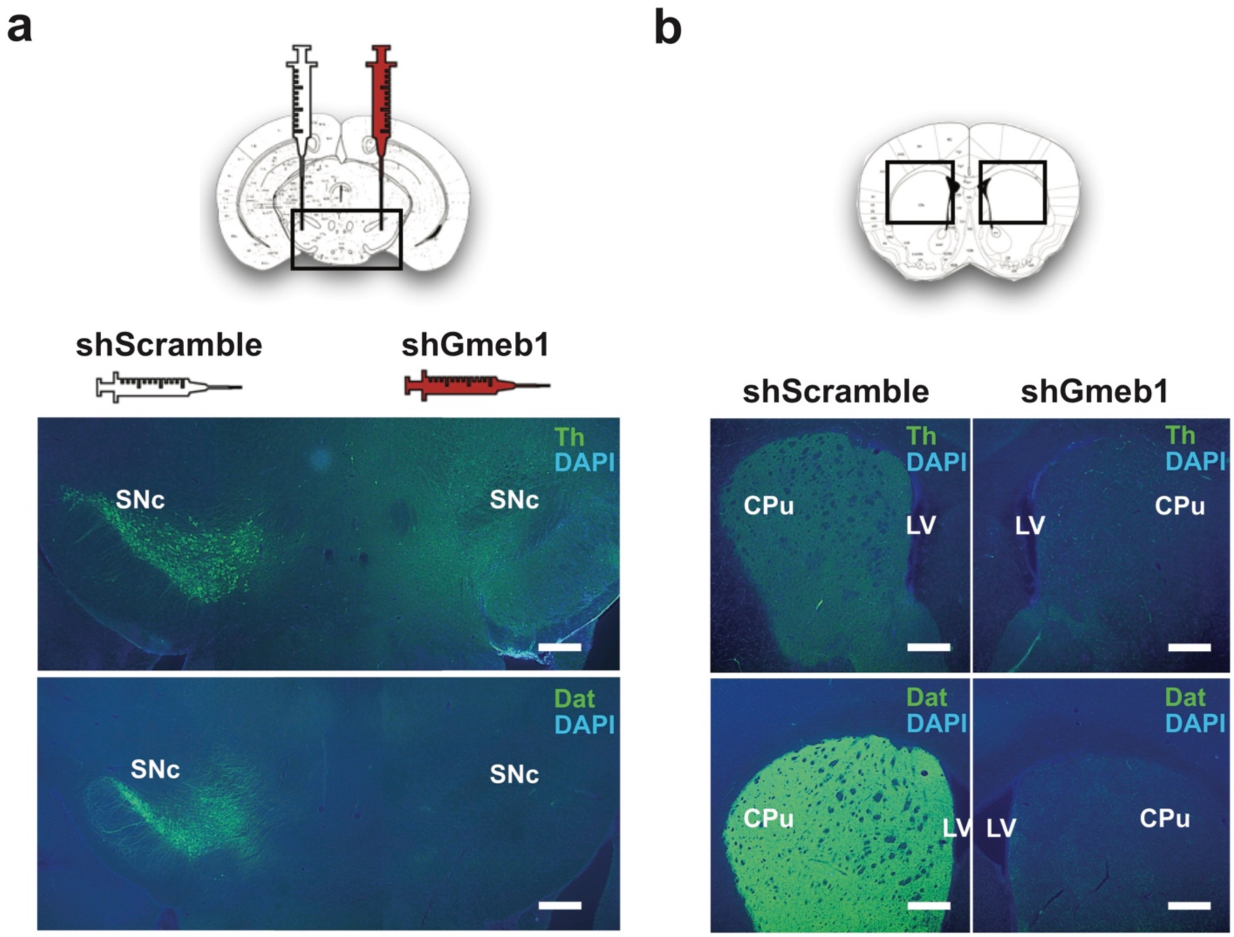

Supplementary Figure 8 | (Related to Figure 3) *Gmeb1* knockdown in SNc depletes Th and Dat in SNc and CPu

- **(a)** 2.5x midbrain micrographs showing Th/DAPI stain (top) and Dat/DAPI stain (bottom) 2 weeks following unilateral SNc injection of AVV5-DIO-KASH-GFP-U6-shGmeb1 and contralateral shScramble control injection. Vertical syringes indicate location of virus injections. Box insert reflects brain region where micrograph was obtained. Scale bar: 500 µm. Note: Needle/syringe image was adapted from Keynote clipart, and coronal brain section was reproduced from **Ref. 48** (Copyright 2013, Elsevier, Academic Press).
- **(b)** 2.5x CPu micrographs showing Th/DAPI stain (top) and Dat/DAPI stain (bottom) 2 weeks following unilateral SNc injection of AVV5-DIO-KASH-GFP-U6-shGmeb1 and contralateral shScramble control injection. Box inserts reflect brain region where micrographs were obtained. Scale bar: 500 µm. Note: Coronal brain section was reproduced from **Ref. 48** (Copyright 2013, Elsevier, Academic Press).

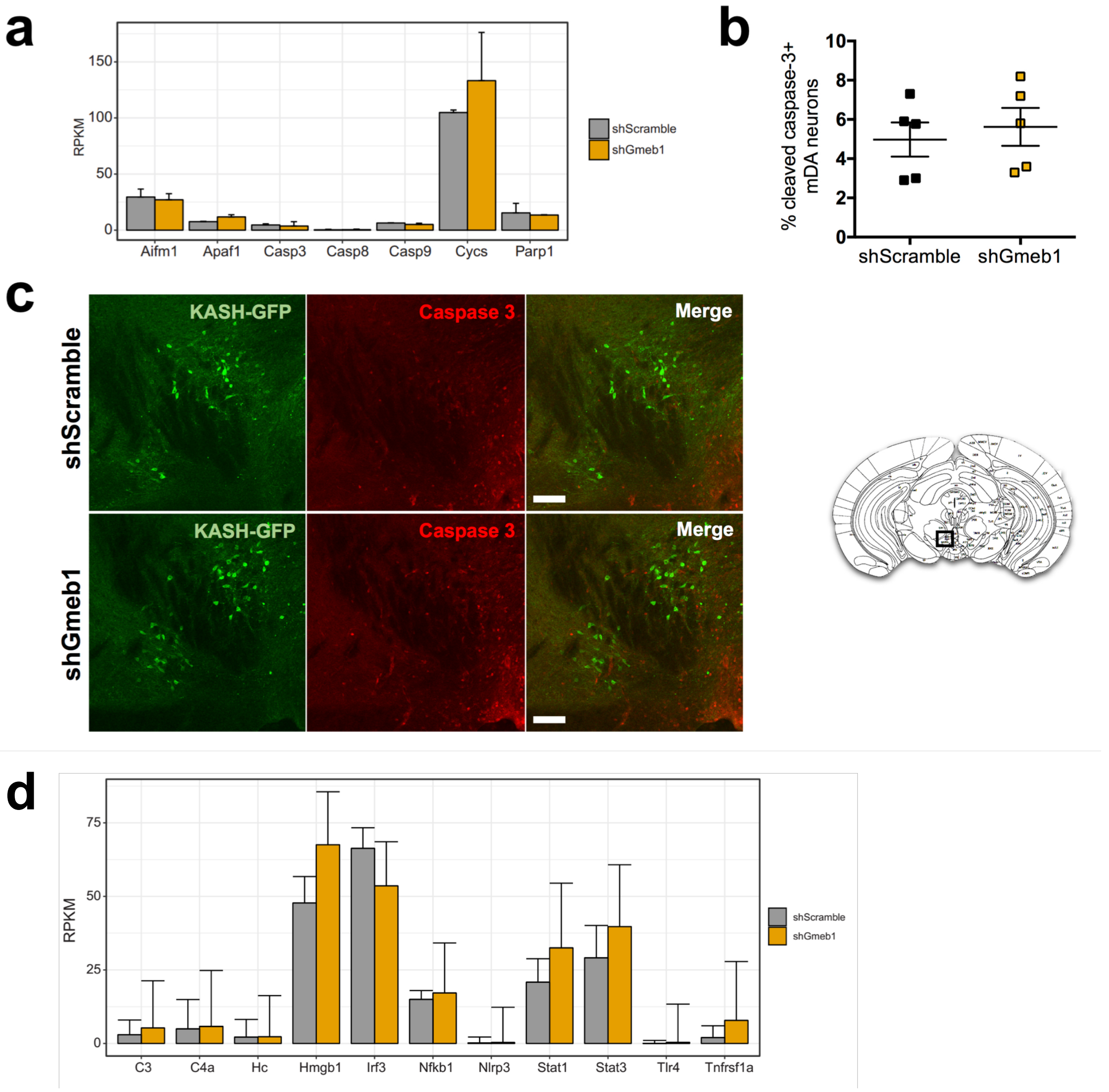

Supplementary Figure 9 | (Related to Figure 3) *Gmeb1* knockdown in midbrain does not induce expression of apoptosis or inflammation markers.

- **(a)** Bar graphs showing the mean (± s.e.m.) FPKM of apoptosis-relevant genes in shScramble-treated (grey) or shGmeb1-treated (orange) mDA neurons.
- **(b)** Dot plot showing mean (± s.e.m.) percentage of mDA neurons (as judged by KASH-GFP expression) that co-express cleaved caspase 3. Result is not significant: p=0.6323 in student's t-test. (n=5 per group, 6 midbrain slices were examined per animal to obtain cell counts).
- **(c)** Representative micrographs of KASH-GFP-expressing mDA neurons (left panels) co-stained with cleaved caspase 3 (center panels). Gmeb1 knockdown does not cause cell apoptosis as indicated by unchanged cleaved caspase 3 level. Scale bar: 100 µm. Note: Coronal brain section was reproduced from **Ref. 48** (Copyright 2013, Elsevier, Academic Press).
- **(d)** Bar graphs showing the mean (± s.e.m.) FPKM of inflammation-relevant genes in shScramble-treated (grey) or shGmeb1-treated (orange) mDA neurons.

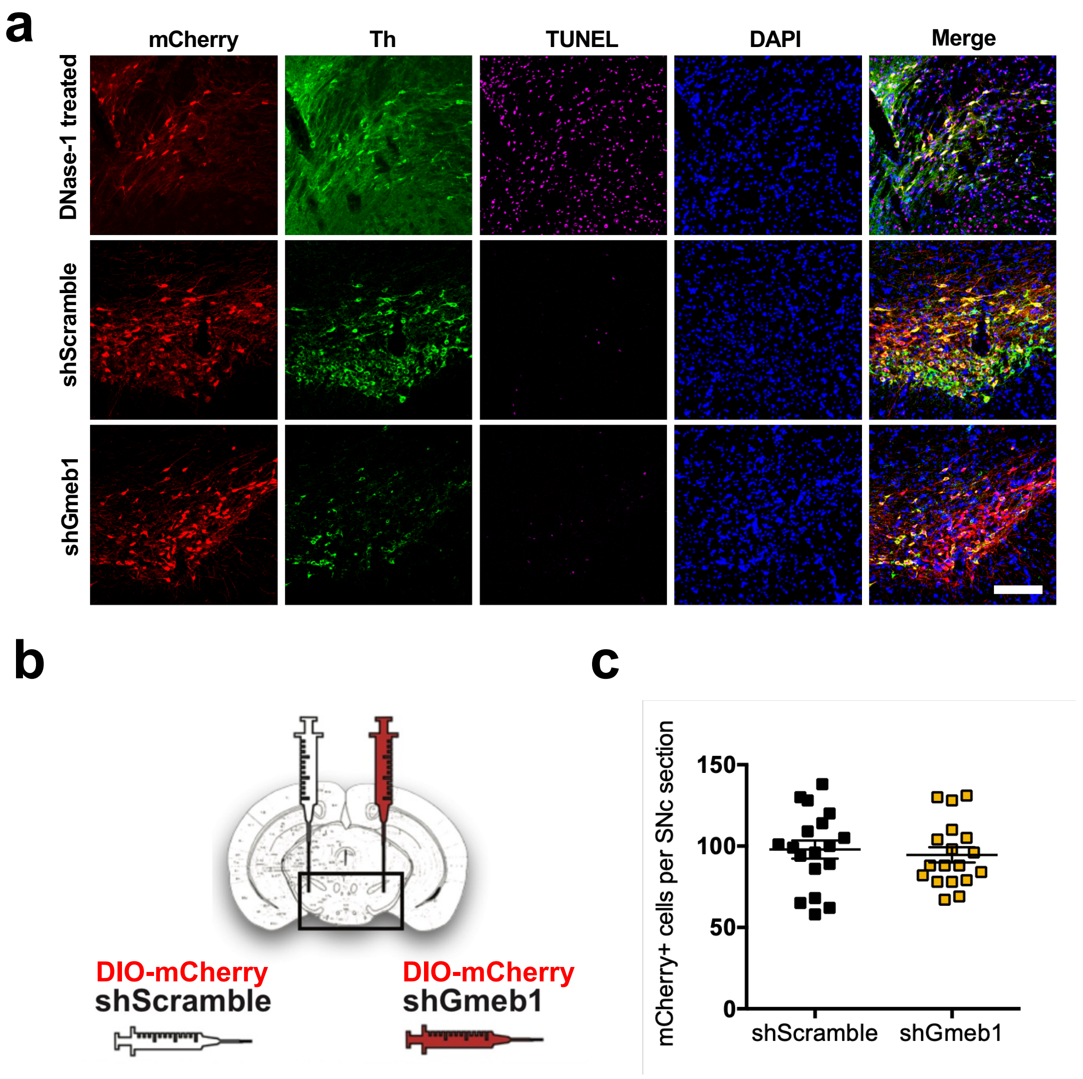

Supplementary Figure 10 | (Related to Figure 3) *Gmeb1* knockdown in midbrain does not result in death of mDA neurons.

- **(a)** Micrographs showing presence of mCherry (red), Th (green), TUNEL (purple), and DAPI (blue) in SNc sections of treated with shGmeb1 (bottom row), shScramble control (middle row) and DNase-1 as a positive control for TUNEL staining (top). DNase-1 treatment shows widespread DNA damage indicative of cell death, whereas control and *Gmeb1* knockdown tissue show lesser amounts of TUNEL signal. Scale bar: 250 µm.
- **(b)** Diagram showing injection strategy for micrographs shown in **(a)**: Dat-Cre mice were co-injected with a 1:1 mix of AAV-DIO-mCherry and either shGmeb1 or shScramble virus on the contralateral SNc. The black squares reflect midbrain region where micrographs were obtained. Note: Needle/syringe image was adapted from Keynote clipart, and coronal brain section was reproduced from **Ref. 48** (Copyright 2013, Elsevier, Academic Press).
- **(c)** Dot plot showing mean (± s.e.m.) number of mCherry+ (DA) neurons in SNc sections 2 weeks following injection as shown in **(b)**. 3 mice were injected per group and 6 SNc sections were analyzed per mouse. No significant difference between the two groups was observed. (n=18 SNc sections, p=0.5364 in student's t-test).

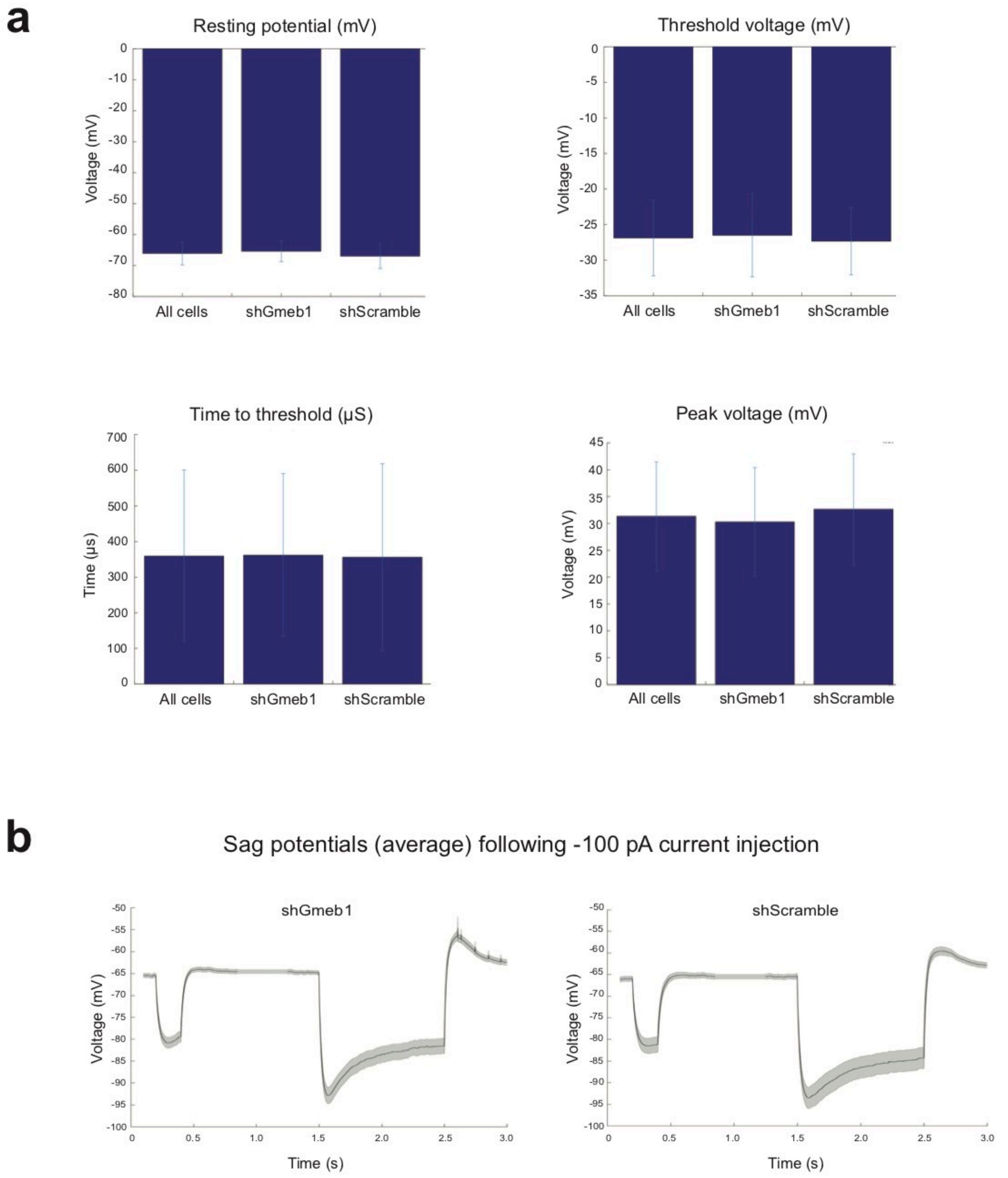

Supplementary Figure 11 | (Related to Figure 4) Baseline electrophysiology of Gmeb1 knockdown and control SNc mDA neurons.

- **(a)** Bar graphs (± s.d.) showing no difference in resting potential, AP threshold voltage, time required to reach AP threshold, and peak AP voltage in SNc mDA neurons containing either knockdown of Gmeb1 (n=26 cells) or shScramble control (n=19 cells).
- **(b)** Average (± s.d.) traces of membrane potential sag in SNc mDA neurons containing either knockdown of Gmeb1 (n=26 cells) or shScramble control knockdown (n=19 cells).

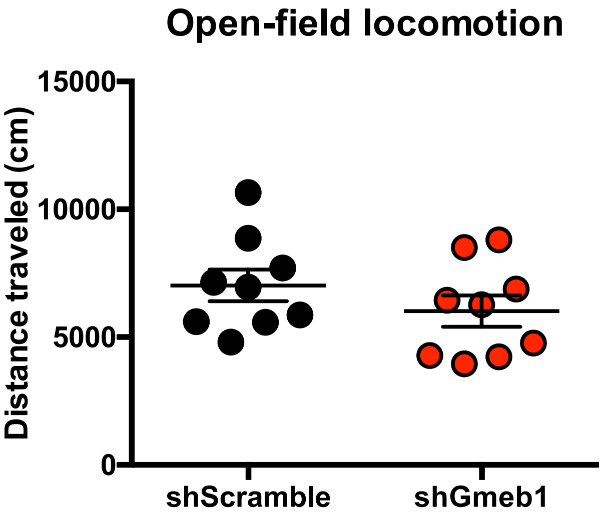

Supplementary Figure 12 | (Related to Figure 5) Open-field locomotion. Mean (± s.e.m.) distance traveled by mice in an open-field arena. Mice were allowed to explore the entirety of an open field arena for 30 minutes during which time the distance traveled (cm) was recorded as beam breaks, and the individual scores were averaged per group (n=9, p=0.2639 in student's t-test).
