# Supplementary material for: In vivo nuclear capture and molecular profiling identifies Gmeb1 as a transcriptional regulator essential for dopamine neuron function
Source: Nat Commun. 2019 Jun 7;10:2508. doi: 10.1038/s41467-019-10267-0 (PMC6555850; doi:10.1038/s41467-019-10267-0)
Supplement: Supplementary file 3 — Reporting Summary [file 41467_2019_10267_MOESM3_ESM.pdf]

Corresponding author(s): Yi Zhang, Ph.D.

Life Sciences Reporting Summary

Nature Research wishes to improve the reproducibility of the work that we publish. This form is intended for publication with all accepted life science papers and provides structure for consistency and transparency in reporting. Every life science submission will use this form; some list items might not apply to an individual manuscript, but all fields must be completed for clarity.

For further information on the points included in this form, see Reporting Life Sciences Research. For further information on Nature Research policies, including our data availability policy, see Authors & Referees and the Editorial Policy Checklist.

Please do not complete any field with "not applicable" or n/a. Refer to the help text for what text to use if an item is not relevant to your study. For final submission: please carefully check your responses for accuracy; you will not be able to make changes later.

Experimental design

1. Sample size

Describe how sample size was determined.

Animal sample size was determined by previous published empirical data and preliminary behavioral experiments.

2. Data exclusions

Describe any data exclusions.

No animals were excluded in this study. However, standard exclusion criteria include:
-Poor body condition due to treatment/surgery. In this case, animals are euthanized and all data are excluded from analysis.
-Virus injection is found to be anatomically off-target during post-mortem histological verification.

3. Replication

Describe the measures taken to verify the reproducibility of the experimental findings.

For all experiments, all attempts at replication were successful.

4. Randomization

Describe how samples/organisms/participants were allocated into experimental groups.

Mice housed in the same cage were randomly assigned to experimental groups, and mice representing each experimental group were evenly distributed among testing sessions.

5. Blinding

Describe whether the investigators were blinded to group allocation during data collection and/or analysis.

Whenever possible, the experimenter was blind to experimental and/or treatment group.

Note: all in vivo studies must report how sample size was determined and whether blinding and randomization were used.

6. Statistical parameters

For all figures and tables that use statistical methods, confirm that the following items are present in relevant figure legends (or in the Methods section if additional space is needed).

n/a	Confirmed	
☐	☒	The exact sample size (*n*) for each experimental group/condition, given as a discrete number and unit of measurement (animals, litters, cultures, etc.)
☐	☒	A description of how samples were collected, noting whether measurements were taken from distinct samples or whether the same sample was measured repeatedly
☐	☒	A statement indicating how many times each experiment was replicated
☐	☒	The statistical test(s) used and whether they are one- or two-sided<br>*Only common tests should be described solely by name; describe more complex techniques in the Methods section.*
☒	☐	A description of any assumptions or corrections, such as an adjustment for multiple comparisons
☐	☒	Test values indicating whether an effect is present<br>*Provide confidence intervals or give results of significance tests (e.g. P values) as exact values whenever appropriate and with effect sizes noted.*
☐	☒	A clear description of statistics including central tendency (e.g. median, mean) and variation (e.g. standard deviation, interquartile range)
☐	☒	Clearly defined error bars in all relevant figure captions (with explicit mention of central tendency and variation)

See the web collection on statistics for biologists for further resources and guidance.

Software

Policy information about availability of computer code

7. Software

Describe the software used to analyze the data in this study.

- GraphPad Prism 6 was used for statistical analyses of behavioral studies and histological cell counts.
- Trimmomatic (v.0.36) was used for read trimming.
- STAR (v.2.5.2b) used for RNA-Seq data mapping.
- RSEM (v1.2.31) was used for gene expression quantification.
- Bowtie2 (v2.2.9) was used for DNA-Seq data mapping.
- Macs2 (v2.1.1.20160309) was used for peak calling.
- Samtools (v.1.3.1) was used for bam and sam file manipulation.
- Bpipe (v0.9.9.2) for the pipelines automation.
- R (v3.3.1) was used for all bioinformatics data analysis.

For manuscripts utilizing custom algorithms or software that are central to the paper but not yet described in the published literature, software must be made available to editors and reviewers upon request. We strongly encourage code deposition in a community repository (e.g. GitHub). *Nature Methods* guidance for providing algorithms and software for publication provides further information on this topic.

Materials and reagents

Policy information about availability of materials

8. Materials availability

Indicate whether there are restrictions on availability of unique materials or if these materials are only available for distribution by a third party.

No restrictions on availability of unique materials.

9. Antibodies

Describe the antibodies used and how they were validated for use in the system under study (i.e. assay and species).

Primary antibodies:
-HA (Cell Signaling, 3724). 1:200 for nuclear capture, 1:800 for floating section staining.
-Th (Santa Cruz, SC-25269). 1:500 for floating section staining.
-GFP (AbCam, Ab-1390). 1:2000 for nuclear capture, 1:2500 for floating section staining.
-Dat (GeneTex, GTX30992). 1:500 for floating section staining.
-Caspase-3 (AbCam, Ab-13847). 1:500 for floating section staining.
-mCherry (AbCam, Ab-167453). 1:500 for floating section staining.

HA, GFP, Dat, Th, Caspase-3, and mCherry antibodies were validated using negative controls (based on Cre expression or by comparison to anatomical region lacking target antigen).

Secondary antibodies:
-Alexa Fluor 568 donkey-anti-rabbit (Invitrogen, A10042)
-Alexa Fluor 488 donkey-anti-mouse (Invitrogen, A21202)
-Alexa Fluor 488 donkey-anti-rat (Invitrogen, A21208)
-TRITC-conjugated donkey-anti-chicken (Jackson Immunoresearch, 703-025-155)
-FITC-conjugated donkey-anti-chicken (Jackson Immunoresearch, 703-095-155)
*Note: All secondary antibodies were used at the same concentration for all applications (1:500).

10. Eukaryotic cell lines

a. State the source of each eukaryotic cell line used.

Neuro-2A cells were purchased from ATCC (CCL-131).

b. Describe the method of cell line authentication used.

Neuro-2A cells obtained were authenticated by ATCC.

c. Report whether the cell lines were tested for mycoplasma contamination.

The cell line was tested for mycoplasma contamination.

d. If any of the cell lines used are listed in the database of commonly misidentified cell lines maintained by ICLAC, provide a scientific rationale for their use.

Provide a rationale for the use of commonly misidentified cell lines OR state that no commonly misidentified cell lines were used.

Animals and human research participants

Policy information about studies involving animals; when reporting animal research, follow the ARRIVE guidelines

11. Description of research animals

Provide all relevant details on animals and/or animal-derived materials used in the study.

-For molecular profiling studies, we used 8-12 week-old male DAT-Cre heterozygous mice and wildtype littermates. These were derived from crossing female DAT-Cre heterozygous mice (Jackson Labs, 06660) with male C57BL/6J wildtype mice (Jackson Labs, 000664).
-For electrophysiological studies, we used 8 week-old male DAT-Cre heterozygous mice (Jackson Labs, 06660).
-Only male mice were used for profiling experiments whereas both male and female mice were used for histological analysis and establishment of the infection system.
-For all behavior studies, we used 12-week old male C57BL/6J wildtype mice (Jackson Labs, 000664).

Policy information about studies involving human research participants

12. Description of human research participants

Describe the covariate-relevant population characteristics of the human research participants.

n/a
