# Supplementary material for: In vivo nuclear capture and molecular profiling identifies Gmeb1 as a transcriptional regulator essential for dopamine neuron function
Source: Nat Commun. 2019 Jun 7;10:2508. doi: 10.1038/s41467-019-10267-0 (PMC6555850; doi:10.1038/s41467-019-10267-0)
Supplement: Supplementary file 4 — Description of Additional Supplementary Information [file 41467_2019_10267_MOESM4_ESM.pdf]

Description of Additional Supplementary Files

File Name: Supplementary Data 1
Description: List of the 107 mDA-enriched genes.

File Name: Supplementary Data 2
Description: List of the 287 HA+ enriched genes that are not significantly enriched in comparison to cortical neurons.

File Name: Supplementary Data 3
Description: List of the genes enriched in PV, VIP and Exc cortical neurons

File Name: Supplementary Data 4
Description: Matrix of regulatory scores for the 85 distal DHS-containing mDA-enriched genes.

File Name: Supplementary Data 5
Description: Matrix of regulatory scores for the 59 promoter DHS-containing mDA-enriched genes.

File Name: Supplementary Data 6
Description: List of the 99 genes down-regulated by Gmeb1 knockdown in mDA neurons.

File Name: Supplementary Data 7
Description: List of the 78 genes up-regulated by Gmeb1 knockdown in mDA neurons.

File Name: Supplementary Data 8
Description: Summary of the datasets generated in this study.

File Name: Supplementary Movie 1
Description: Pole test. Sample video of SNc shScramble (left) and shGmeb1 (right) knockdown mice completing the pole test. The time taken to climb down to the base of the pole was measured.

File Name: Supplementary Movie 2
Description: Rotarod test. Sample video of SNc shScramble (left) and shGmeb1 (right) knockdown mice completing the rotarod test. Latency was calculated as the time elapsed until the animal fell from the rod or grasped on for 1 revolution without trotting.

File Name: Supplementary Movie 3
Description: Swim test. Abridged sample video of SNc shScramble (left) and shGmeb1 (right) knockdown mice completing the swim test. Swimming dexterity was scored on a scale of 0-5 with the higher score indicating greater swimming dexterity.

File Name: Supplementary Movie 4
Description: Hanging wire test. Sample video of SNc shScramble (left) and shGmeb1 (right) knockdown mice completing the hanging wire test. Scores were calculated based on the time elapsed until animal fell from wire onto the padded surface.
